# Achieving consistency of flexible surface acoustic wave sensors with artificial intelligence

**DOI:** 10.1038/s41378-024-00727-z

**Published:** 2024-07-05

**Authors:** Zhangbin Ji, Jian Zhou, Yihao Guo, Yanhong Xia, Ahmed Abkar, Dongfang Liang, Yongqing Fu

**Affiliations:** 1 College of Mechanical and Vehicle Engineering, Hunan University, Changsha, 410082 China; 2 Department of Engineering, University of Cambridge, Trumpington Street, Cambridge, CB2 1PZ UK; 3 Faculty of Engineering and Environment, Northumbria University, Newcastle upon Tyne, NE1 8ST UK

**Keywords:** Electrical and electronic engineering, Materials science, Physics

## Abstract

Flexible surface acoustic wave technology has garnered significant attention for wearable electronics and sensing applications. However, the mechanical strains induced by random deformation of these flexible SAWs during sensing often significantly alter the specific sensing signals, causing critical issues such as inconsistency of the sensing results on a curved/flexible surface. To address this challenge, we first developed high-performance AlScN piezoelectric film-based flexible SAW sensors, investigated their response characteristics both theoretically and experimentally under various bending strains and UV illumination conditions, and achieved a high UV sensitivity of 1.71 KHz/(mW/cm²). To ensure reliable and consistent UV detection and eliminate the interference of bending strain on SAW sensors, we proposed using key features within the response signals of a single flexible SAW device to establish a regression model based on machine learning algorithms for precise UV detection under dynamic strain disturbances, successfully decoupling the interference of bending strain from target UV detection. The results indicate that under strain interferences from 0 to 1160 με the model based on the extreme gradient boosting algorithm exhibits optimal UV prediction performance. As a demonstration for practical applications, flexible SAW sensors were adhered to four different locations on spacecraft model surfaces, including flat and three curved surfaces with radii of curvature of 14.5, 11.5, and 5.8 cm. These flexible SAW sensors demonstrated high reliability and consistency in terms of UV sensing performance under random bending conditions, with results consistent with those on a flat surface.

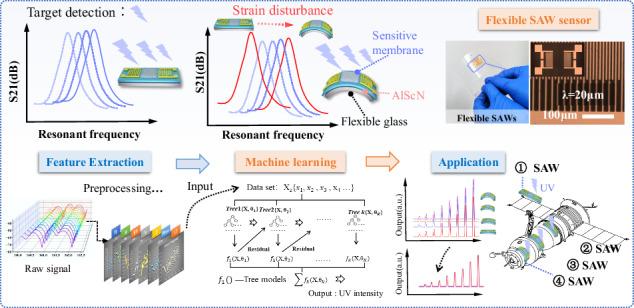

## Introduction

**Fig. 1:**
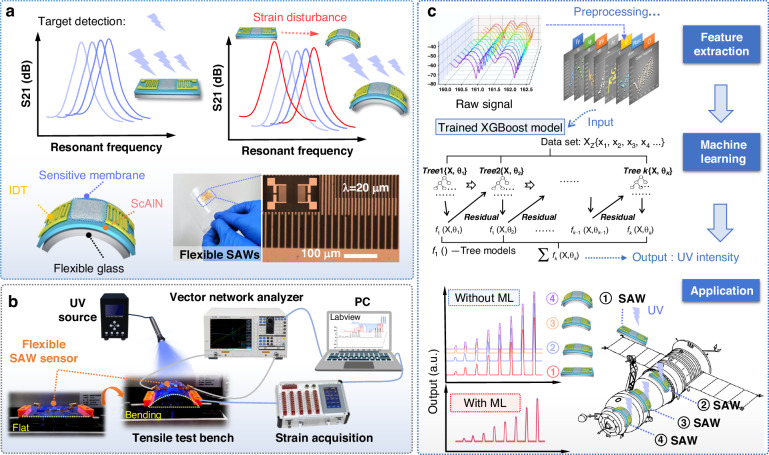
Machine learning-enabled consistent detection on a flexible surface acoustic wave platform under random bending conditions. **a** schematic view of the AlScN/glass-based flexible SAW device and detection of target parameters under bending disturbance; **b** schematic diagram of the testing configuration of the SAW device; **c** machine learning model used for eliminating bending interference

Surface acoustic wave (SAW) technology has been widely applied in fields such as communication systems, microfluidics, acoustic tweezers, quantum acoustics, and single-electron control^1–5^. In particular, because of its wireless/passive operation and compact design capability, it is highly suitable for various sensing applications^6–9^. Recently, flexible or bendable SAW devices have gained significant attention because they offer many new applications compared to rigid and conventional devices^10–13^. These flexible SAW devices possess distinctive characteristics, such as light weight, good biocompatibility, and adjustable mechanical conformability to object surfaces, showing their great potential for signal monitoring on curved surfaces. Various types of flexible SAW devices have been developed over the past decade^6,14–22^, and significant progress has been made in flexible physical/biochemical sensors with good sensing performance^6,21,23–25^. Flexibility and bendability are two key characteristics of flexible SAW sensors. However, a key challenge for these flexible SAW devices is that they experience significant frequency shifts or signal interference when they are subjected to different bending conditions (or under various mechanical strains), primarily due to changes in the acoustic wave velocities (acoustic elastic effects) and deformation of the interdigital transducers (IDTs)^26^. These signal changes are induced not by the targeted sensing information but by the applied strains or deformation compared to those in the planar state. Such strain induced frequency shifts strongly interfered with the targeted sensing signals (such as ultraviolet light signals, gas molecules, or biological species), leading to reduced accuracy or stability of these flexible SAW sensors (Fig. 1a). Reference samples are typically employed to mitigate the influences of this deformation and bending strain, yet this approach complicates the entire sensing platform.

In pursuit of a reliable and accurate flexible acoustic wave sensing system, our research team previously implemented an off-axis design that minimized the bending strain effects for AlN/ultrathin glass flexible SAW devices^27^. This device-level design methodology was employed to mitigate signal variations induced by bending strains, enabling the extraction of signals solely originating from the intended monitoring parameter. However, despite its effectiveness, this approach has certain limitations, such as stringent requirements for the film deposition quality, wavelength, and film thickness ratio for the SAW device, thereby impeding its widespread applicability. Therefore, new methods for eliminating the signal changes induced by the bending strain of these flexible SAW devices and obtaining only the monitored signals of the target parameters are urgently needed. However, few studies have been conducted to address this challenge.

In this paper, we developed a flexible SAW sensor based on AlScN thin films deposited on an ultrathin flexible glass substrate and then investigated the effects of strain and ultraviolet (UV) light on the sensing characteristics of flexible SAW devices (Fig. 1b). Additionally, the influence of bending strain on the performance of the SAW device was analyzed through theoretical calculations. We introduced machine learning algorithms to establish a regression model that correlates the response signal features of the SAW device with the targeted sensitive parameter (e.g., UV intensity as an example in this study) under dynamic strain perturbations (Fig. 1c). A flexible SAW sensing platform was implemented based on this model to eliminate strain interference. We also compared the comprehensive predictive performance of models constructed using various machine learning algorithms, including multiple linear regression (MLR), polynomial regression (PR), ridge regression (Ridge), robust regression (Robust), elastic net regression (ENR), decision trees (DTR), random forests (RFR), and extreme gradient boosting (XGBoost). Furthermore, SHapley Additive exPlanations (SHAP) values were utilized to interpret and investigate the influences of patterns of input feature variables on UV intensity prediction under various dynamic straining conditions. Finally, we validated the generalizability of the model using a randomly selected validation set, conducted a conceptual demonstration on a scaled spacecraft model, and proved the consistency of the UV detection results for different curved surfaces and planes. Our developed flexible UV SAW sensors with machine learning algorithms open new avenues for various innovative applications. For example, in aerospace applications, these UV sensors can be strategically placed on the curved surfaces of aircraft or satellites to monitor UV radiation, which is essential for understanding space weather effects and ensuring the longevity of spacecraft materials.

## Methods

### Fabrication of flexible SAWs

AlScN films (with a Sc concentration of ~40%) were deposited onto ultrathin and flexible glass substrates (100 μm thick) using magnetron sputtering techniques. The interlayers were not employed in this film fabrication process. Before the sputtering process, the substrate surface was cleaned and activated by nitrogen ion bombardment to ensure that the high-energy surface was ready for the growth of the AlScN thin films. A 99.99% purity aluminum-scandium (AlSc) alloy target with a mass ratio of Al:Sc (0.6:0.4) and oxygen impurities below 500 ppmw was used for reactive magnetron sputtering. During the deposition, a gas mixture of nitrogen (99.999%) and argon (99.999%) was introduced at a flow rate of 30/20 sccm, with a gas pressure of 0.15 Pa. The substrate temperature was controlled at 450 °C, and the substrate was rotated at a speed of 20 revolutions per minute to ensure uniform film deposition. A pulsed direct current power of 10 kW was used, along with a radio frequency bias power of 160 W, with a power supply frequency ranging from 100–250 kHz to enhance the film’s crystallinity and adhesion to the substrate.

Characterization of the crystal orientations of the AlScN films was performed using an X-ray diffractometer (PANalytical Empyrean) equipped with a Cu-Kα radiation source and a scanning angle range of 2θ = 5° –90°. The surface morphology and roughness of the films were characterized using an atomic force microscope (AFM, Dimension Icon, Bruker). The cross-sectional morphologies of the prepared films were determined using scanning electron microscopy (SEM, ZEISS Sigma300). The elemental composition and mapping analysis of the AlScN thin films were characterized using an energy dispersive spectroscopy (EDS) analyzer (Aztec X-MaxN 20, Oxford Instruments). The elemental composition, chemical state, and molecular structure of the AlScN films were analyzed using X-ray photoelectron spectroscopy (XPS, Shimadzu AXIS Supra + Japan).

Interdigital transducers (IDTs) were fabricated on AlScN film-coated glass with 10 nm of Cr and 60 nm of Au using standard photolithography and subsequent lift-off processes, and the wavelength was set at 20 μm. The flexible SAW devices were designed with 50 pairs of IDTs with a metallized ratio of 0.5 and an aperture of 200 λ. The center distance between the two IDTs was 200 λ, the SAW devices had 100 pairs of reflectors, and the distance between the reflectors and IDTs was ¼ λ. The scattering (S) parameters were obtained using a network analyzer (3656D, Ceyear). A SAW device was attached to 0.5 mm thick polyethylene terephthalate (PET) using a UV-curable GGJ-1 adhesive, and its resultant good flexibility is demonstrated in Fig. 1a.

### Sensing setup and experiment

To collect UV response data from flexible SAW devices under different strains, SAW devices were subjected to various bending conditions and UV intensities. The SAW device was first bound onto a flexible printed circuit board (PCB) and then to a 0.5 mm thick using an adhesive of UV-curable GGJ-1. A tensile testing machine was used to bend the PET, and different bending strains were applied. This procedure (Fig. S1) is explained as follows. We utilized a pulse controller to regulate the speed of the servo motor. Once the motor was activated, it rotated and drove two blocks (mounted on a lead screw), thus causing them to move uniformly. These blocks were connected to the ends of the PET plate, with the flexible SAW device affixed to the surface of the PET plate. By altering the distance between the two blocks, we induced bending in the flexible SAW devices, which effectively introduced a continuous variation in stress, known as the dynamic strain. Additionally, one standard strain gauge (BF1K-3EB with a full bridge foil strain gauge) was affixed near the SAW sensor to collect the local strain values and calibrate the strain changes. For UV sensing tests (with a wavelength of 365 nm), a 2 mg/mL ZnO nanowire (NW) solution (Xianfeng Company, China) was prepared by adding ZnO-NW powder to deionized water and then drop-cast onto the surface of the SAW device to form a UV-enhanced sensing layer. The coated ZnO-NW-sensitive layer was used because it exhibited a significant improvement in UV sensing performance (approximately tenfold enhancement in both sensitivity and response speed) compared to that of the uncoated devices (Fig. S2). The changes in the S_21_ spectra of the devices in different states were recorded using a network analyzer, and the LabVIEW program was used to determine the time-domain signal changes of the SAW sensors.

## Results and discussion

### AlScN film characterization and flexible SAW devices

**Fig. 2:**
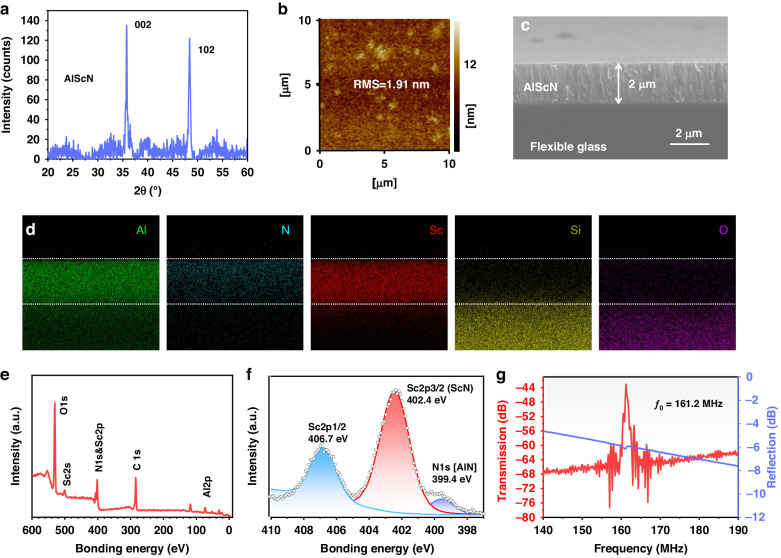
Characterization of AlScN/ultrathin flexible glass. **a** XRD pattern, **b** AFM image, and **c** cross-sectional SEM image of the AlScN film; **d** elemental maps, **e** XPS survey spectrum, and high-resolution spectra of (**f**) N1s and Sc2p of AlScN/ultrathin flexible glass; **g** scattering parameters of the AlScN/glass-based flexible SAW device

The AlScN thin film exhibits a strong (002) crystal orientation, as evidenced by the presence of a diffraction peak at a 2θ angle of 35.78° in its XRD spectrum(Fig. 2a). Additionally, there is another peak corresponding to the AlScN (102) crystal plane at 48.4°, which is possibly formed due to crystal formation due to the amorphous properties of the flexible glass substrate during the sputtering process. The surface characteristics of the film, as illustrated in Fig. 2b, exhibit a smooth root-mean-square roughness (RMS) of ~1.91 nm across a 10 × 10 μm² area. The SEM image of the AlScN film is presented in Fig. 2c, illustrating the columnar structures of the AlScN nanocrystals, which are oriented perpendicularly to the substrate, with an average thickness of 2 μm. The EDS analysis results, as shown in Fig. 2d, indicate that the Al, Sc, and N in the AlScN thin films are uniformly distributed. The weight percentages of N, Al, and Sc are 31.13 wt.%, 31.80 wt.%, and 37.07 wt.%, respectively. The N1s spectrum shown in Fig. 2f displays two distinct binding energy components after peak deconvolution with a curve-fitting program. These peaks are located at binding energies of 399.04 eV and 402.40 eV, signifying the presence of Al-N and Sc-N bonds, respectively.

Figure 2g shows the reflectance (S_11_) and transmittance (S_21_) spectra of the AlScN/ultrathin glass flexible SAW device. A pronounced Rayleigh resonance peak was identified, corresponding to a resonance frequency of 161.2 MHz, with an S_21_ signal amplitude up to 25 dB. Compared to the previously reported AlN/ultrathin glass flexible SAW devices at a similar range of wavelengths^22^, a slight decrease in the resonance frequency was observed in the AlScN/ultrathin glass flexible SAW device. This decrease is attributed to the softening of the piezoelectric film caused by the doping of scandium, leading to a decrease in the acoustic wave velocity. The measured electromechanical coupling coefficient of the fabricated flexible SAW device was 1.22%, approximately three times greater than that of the AlN/ultrathin glass flexible SAW device (which was measured as ~ 0.4%)^16^.

### Analysis of flexible SAW bending and UV sensing

**Fig. 3:**
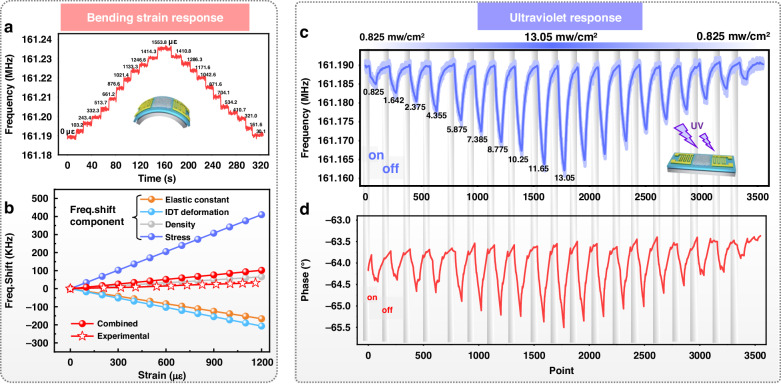
Characterization of bending deformation and UV sensing in an AlScN/glass flexible SAW device. bending deformation and UV sensing characteristics of the AlScN/glass-based flexible SAW device: (**a**) response curves of the flexible SAW device during a strain loading‒unloading cycle; (**b**) experimental and theoretical calculated frequency shifts under different strain states; (**c**, **d**) the frequency and phase characteristics of the flexible SAW sensor change with the UV intensity

For sensing applications, SAW devices are frequently required for application on curved surfaces for sensing or under various strains. Therefore, we first investigated the influences of different strains caused by the bending of the SAW device on the resonance frequencies. Figure 3a illustrates the responses of the flexible SAW device (*λ* = 20 μm) to the applied strain, which increased from 0 to 1553.8 με and then returned to the initial state. The frequency changes show a highly linear relationship between the frequency shift and the applied strain (*R*^*2*^ is ~0.99). Moreover, within a single cycle of frequency-strain recovery, the maximum value of hysteresis in the flexible SAW device is <0.5%. These findings highlight the good performance and stability of the developed flexible SAW device, making it a promising candidate for strain sensing applications. Furthermore, it is important to highlight that the resonant frequency of such AlScN/ultrathin glass flexible SAW devices increases with increasing bending strain, which is quite different from the responses observed in previous studies using flexible SAW devices based on polymers (such as polyethylene terephthalate and polyethylene naphthalate) or aluminum foil substrates^17,28,29^.

To unravel the underlying mechanisms, such as the bending responses of AlScN/glass flexible SAW devices, we performed theoretical calculations to discern the relationship between frequency and strain in the AlScN/glass flexible device (*λ* of 20 μm). The shifts in resonant frequencies observed in the SAW devices under various bending strains are predominantly driven by alterations in wave velocity, induced by fluctuations in the initial stress, elastic constants, and medium density. Additionally, changes in the device wavelength, mainly caused by the deformation of the IDTs, play a significant role. We considered the collective influences of the above factors and computed the frequency-strain responses of the SAW devices. The theoretical values calculated from the fit closely match the experimental results (Fig. 3b). Further information on the analysis details and methods can be found in our previous study^22,27^.

Figure 3b illustrates the calculated frequency shifts resulting from the changes in initial stress, density, elastic constant, device wavelength, and the combined effects of all these factors as a function of bending strain. IDT deformation induces a negative frequency shift, whereas the combined effect of IDT deformation and SAW velocity changes causes a positive frequency shift, clearly indicating that the frequency shift is primarily driven by variations in SAW velocity. Moreover, initial stress and density variations are associated with positive frequency shifts, whereas changes in elastic constants can induce negative frequency shifts. Evidently, modifications in acoustic velocities are contingent upon the combined effects of these three variables, namely, the initial stress, density, and elastic constants. These interactions potentially cause either positive or negative shifts in frequencies. For AlScN/glass flexible SAW devices, the initial stress is the dominant factor governing the frequency-strain shift.

When designing a flexible sensing platform that is resilient to strain interference, a target sensitive parameter must be chosen. In this study, we selected UV intensity as an example and explored the responses of flexible SAW sensors in relation to UV intensity. Figure 3c, d and Figure S3 illustrate the changes in the resonance frequency, phase angle, and insertion loss of the SAW device (*λ* = 20 μm) under various UV intensities. The trends clearly demonstrate that as the ultraviolet (UV) intensity increases, the resonance frequency shift and phase angle deviation of the resonant mode increase. The UV responses of SAW devices are primarily attributed to the acoustoelectric effect. When exposed to UV irradiation, numerous electron-hole pairs, or free carriers, are generated. These carriers then interact with the acoustic wave field, resulting in alterations in the transmission characteristics. These changes encompass shifts in the acoustic wave velocity, variations in the wave amplitude attenuation, and phase angle adjustments^8,30^. The fabricated flexible SAW devices exhibit excellent UV sensing characteristics, with a calculated UV sensitivity of 1.71 KHz/(mW/cm²), a coefficient of determination of 0.997 and hysteresis under 5%.

### UV sensing of flexible SAWs under strain-bending states

**Fig. 4:**
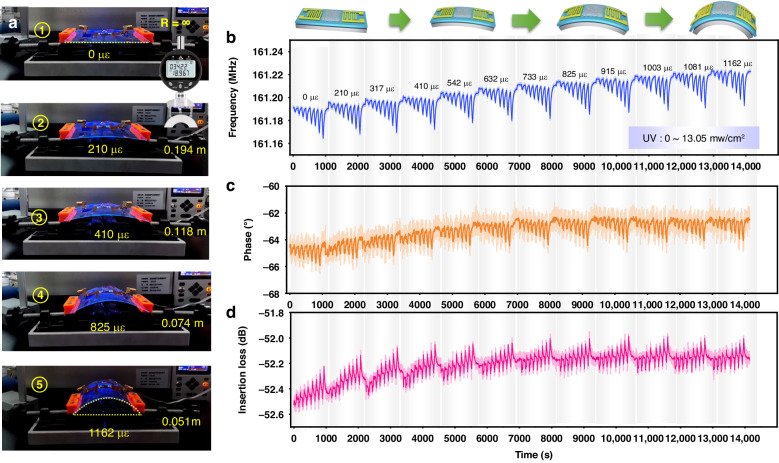
UV sensing of flexible SAWs under strain bending states. **a** photographs of flexible SAW sensors positioned on surfaces with five different curvatures; the response of (**b**) frequency, (**c**) phase and (**d**) insertion loss for flexible SAW devices under various UV intensities and strains

UV sensing tests were performed to collect response data of the flexible SAW devices under various applied strains. Our testing procedure involved applying random bending conditions to flexible SAW sensors to mimic the unpredictable application conditions encountered in real-world scenarios. Figure 4b–d show the changes in the resonance frequency, phase angle and insertion loss of the flexible SAW device under different strain states (0-1,162 με), demonstrating that both the UV and bending strain affect the characteristics of the SAW devices and interfere with each other. By applying wavelet denoising to the collected data^31^, we extracted a set of features, as shown in Fig. 4b–d and Fig. S4, which are composed of the center frequency (Fc), insertion loss (IL), bandwidth (BW), minimum cutoff frequency (Fmin), minimum amplitude (Amin), phase (P), and quality factor (Q). These features, along with their respective labels, constituted the sample dataset for the machine learning program. Subsequently, a random sampling approach was employed, with 70% of the dataset allocated for model training and the remaining 30% utilized for testing purposes. All machine learning regression, statistical analysis, and data mining operations were conducted using open-source Python libraries (i.e., scikit-learn). Prior to training the machine learning models, the input (i.e., seven features extracted from scattering parameters) and output parameters (i.e., ultraviolet intensity) were well defined. The training and validation datasets were composed of 494 distinct input‒output feature pairs, with a total of 20,190 data points. More details of the regression algorithms used are provided in the supplementary materials.

To predict the UV intensity under dynamic strains, 8 models were established based on two classes of machine learning algorithms, including linear regression classes (e.g., MLR, PR, Ridge, Robust, and ENR) and tree model-based regression algorithms (e.g., DTR, RFR, and XGBoost), using 7 features, along with their two corresponding state labels (i.e., the strain and UV intensity).

Three evaluation criteria were established to compare the predictive abilities of various models. The first is the normalized root mean square error (NRMSE), which is normally applied to monitor deviations between model-predicted values and experimentally obtained values. A smaller NRMSE indicates a better prediction performance, which is defined as:

1 $${NRMSE}=\frac{\sqrt{\frac{1}{n}{\sum }_{i=1}^{n}{\left({X}_{{pre},i}-{X}_{{obs},i}\right)}^{2}}}{{X}_{{obs},\max }-{X}_{{obs},\min }}$$

where X_pre,i_ and X_obs,i_ are the predicted value and true value (i.e., obtained outputs) of the ith sample, respectively, and *n* is the total number of datasets. The second is the determination coefficient (*R*^*2*^), which is related to the accuracy of the predicted values relative to the observed values.

2 $${R}^{2}=1-\frac{{\sum }_{i=1}^{n}{\left({X}_{{pre},i}-{X}_{{obs},i}\right)}^{2}}{{\sum }_{i=1}^{n}{\left({X}_{{obs},i}-{\bar{X}}_{{obs}}\right)}^{2}}$$

where ${\bar{X}}_{{obs}}$ is the mean value of the observed outputs. When *R*^*2*^ is near 1, the model has the best prediction capability. The model running time is another evaluation indicator, and the smaller its value is, the greater the efficiency of model operation.

**Fig. 5:**
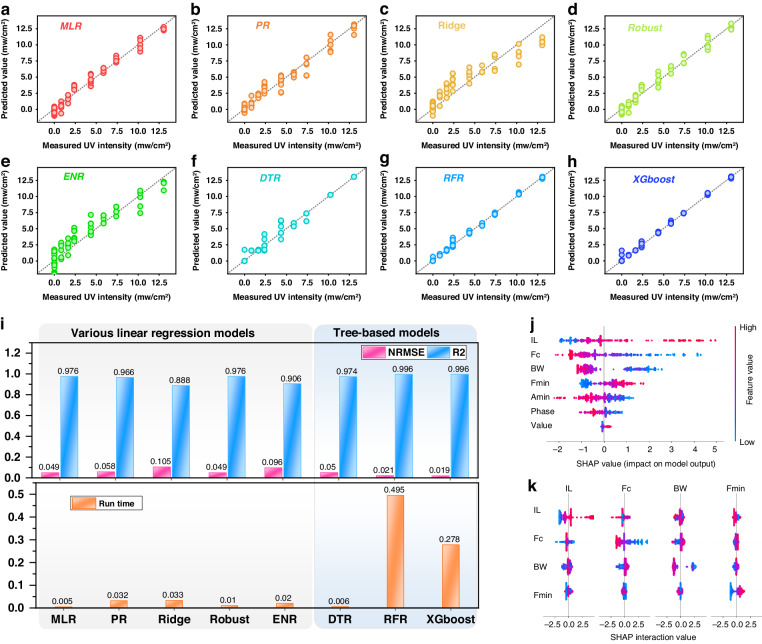
Comprehensive analysis of UV intensity prediction models. The results of prediction of UV intensity by different models, including (**a**) MLR, (**b**) PR, (**c**) Ridge, (**d**) Robust, (**e**) ENR, (**f**) DTR, (**g**) RFR, and (**h**) XGBoost; (**i**) comparisons of comprehensive performance for UV intensity predicted by eight models: prediction accuracy and running time; (**j**) results of overall visualization of features using SHAP values and (**k**) analysis of interactions among multiple variables

Figure 5a–h illustrate the prediction results for the ultraviolet (UV) intensities under dynamic bending strains, which were obtained by employing the model trained using different machine learning algorithms (including MLR, PR, Ridge, Robust, ENR, DTR, RFR, and XGBoost). For the test data shown in the graph, the *x*-axis represents the actual calibrated UV intensity values, and the y-axis represents the model’s output values. The closer the test result points are to the diagonal line (*y* = *x*), the better the predictive performance of the model. Figure 5a–h shows that tree-based models, especially ensemble models such as random forest or extreme gradient boosting, significantly outperform linear regression models.

To quantitatively compare the overall predictive performance of the different models, we calculated the three evaluation metrics mentioned above, and all the results are displayed in Fig. 5i. Although the ensemble models require longer runtime than the linear regression models, the longest runtime required for training and testing all the samples is <0.5 s. The time consumed by these models is negligible for practical sensing applications. Therefore, the runtimes of the models built using the eight machine learning algorithms meet the requirements of practical applications.

In terms of the normalized root mean square error (NRMSE) and determination coefficient (*R*^*2*^), the three models, particularly the XGBoost ensemble model, demonstrate higher prediction accuracy, with the smallest error (NRMSE = 0.019) and the highest *R²* value of 0.996, compared to those of the linear models, as illustrated in Fig. 5i.

While achieving accurate predictions is important for sensor applications, it is equally crucial to understand the rationale behind a model’s predictions. However, attaining the highest accuracy often requires the use of complex models (such as the XGBoost ensemble model, which has demonstrated superior performance in predicting UV intensity). To address this issue, we employed the Shapley additive explanation (SHAP) values proposed by Lundberg and Lee, which is a widely applicable method for explaining predictions^32^. The primary advantage of SHAP values, compared to previous feature importance methods used in the literature^33^, is their ability to reflect the influence of each feature within every sample and indicate which direction it will influence. In Fig. 5j, the x-axis represents the SHAP value, and each row represents a feature, where each point represents a sample with the sample’s color ranging from red (indicating a larger feature value) to blue (indicating a smaller feature value). We can visually observe that the center frequency (Fc) and insertion loss (IL) are two important features. The center frequency is negatively correlated with ultraviolet intensity, whereas the magnitude (or the amplitude) shows the opposite relationship. The interactions among multiple variables are depicted in Fig. 5k, and Figure S5 illustrates the impact of the interaction between the two most important features (i.e., center frequency Fc and insertion loss IL) on the prediction of ultraviolet intensity under dynamic strain. The results demonstrate an approximately linear and negative correlation between the center frequency Fc and the target variable. In contrast, the relationship between the insertion loss (IL) and the target variable exhibits pronounced nonlinearity. Furthermore, since the modes of strain impacting these two features differ from those influenced by ultraviolet radiation, the interaction between these features in the model enhances the accuracy of predicting ultraviolet intensity under dynamic strain conditions. Overall, these results clearly indicate that the existing single feature-based identification strategy is insufficient for accurately predicting ultraviolet intensities under dynamic strains.

### Proof-of-concept for sensing applications

**Fig. 6:**
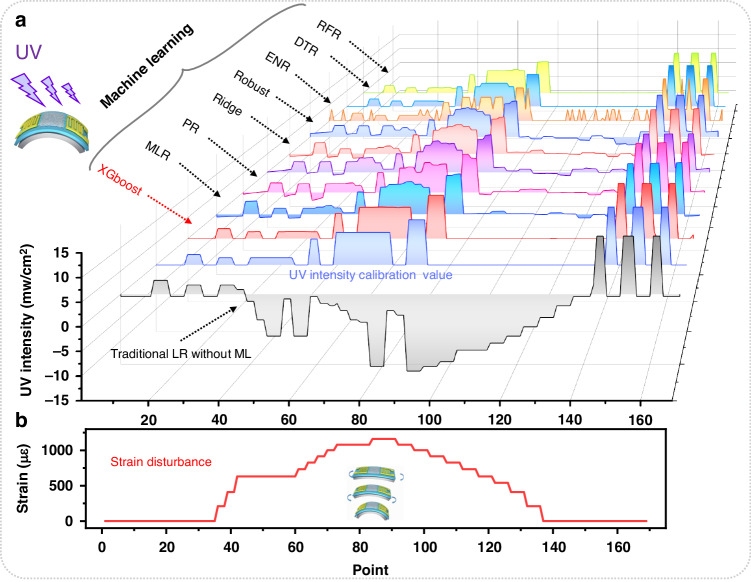
UV intensity prediction under dynamic strain. **a** Comparison of prediction results on the UV sensing test set between conventional linear regression and machine learning models under random strain perturbations. **b** Random strain disturbance under the corresponding state

To validate the generalizability of the developed model, we carried out a UV intensity test under dynamic straining conditions using the fabricated flexible SAW device. We compared the prediction results of frequency changes under various UV intensities using the traditional linear regression approach (based solely on resonance frequency identification), and eight models were trained with different types of machine learning algorithms. Moreover, the results were compared with the actual test results, and the results are shown in Fig. 6a. There are significant deviations in the prediction of the target parameter (i.e., UV intensity) using the traditional linear regression approach, which relies on fitting a linear function based on the relationship between frequency and UV intensity. This discrepancy is attributed to the interference caused by the dynamic strain, as illustrated in Fig. 6b. In contrast, the training models based on different machine learning algorithms demonstrated dramatically improved predictive performance. Among them, the tree-based ensemble model, specifically the XGBoost model, demonstrates the best predictive performance, which is consistent with the evaluation results presented in the previous section. This finding further proves the effectiveness of the XGBoost model in accurately predicting the target parameter under dynamic strain disturbance in flexible SAW devices.

Additionally, reverse analysis can be achieved, and our model is also capable of predicting strains under UV interference. To demonstrate this capability, we conducted strain predictions using the same test dataset. The predictions are presented in Fig. S6, where they are compared with the actual test values. Similar to the UV intensity predictions, models trained with machine learning algorithms showed significant improvement in strain prediction performance. In particular, the proposed XGBoost algorithm model achieved a normalized root-mean-square error of ~1.54% between the predicted and actual strain values, further validating its robustness and reliability.

**Fig. 7:**
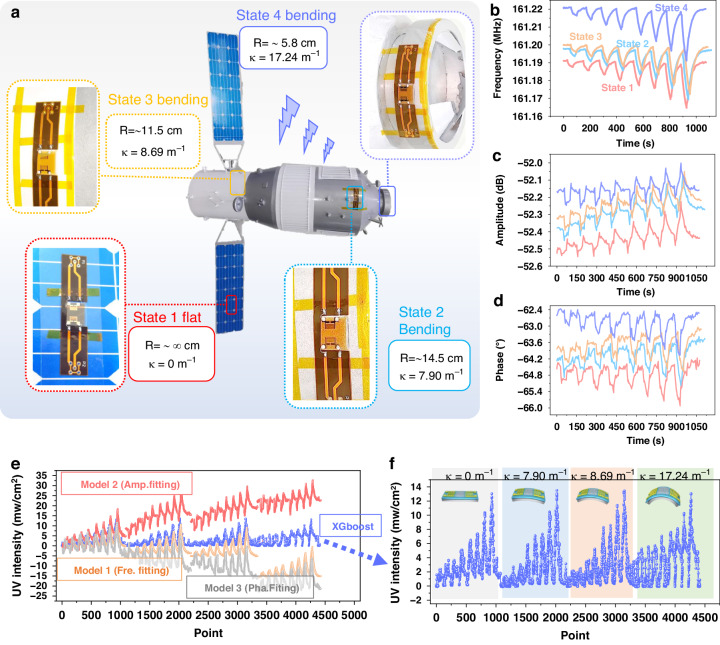
Proof-of-concept for sensing applications. **a** schematic diagram of UV testing using flexible SAW devices, which are arranged on a scaled-down spacecraft casing and include a flat position and three different curved positions with varying curvatures; **b**–**d** signal output results of UV testing for flexible SAW devices in flat and three different curved states, including frequency, amplitude, and phase; **e** comparison of UV output values for flexible devices in four different states using the XGBoost algorithm model and without using a machine learning model (based on single-feature prediction of UV); **f** simplified view of UV prediction results for flexible SAW devices using the ensemble XGBoost algorithm model

Furthermore, we demonstrated the potential aerospace applications of this flexible SAW device with integrated machine learning algorithms for UV sensing. We attached the flexible SAW on the curved surface of a spacecraft model, as shown in Fig. 7a. The flexible SAW sensors were attached at four different and random positions on the surface of the scaled spacecraft model with different bending curvatures, including flat position 1 (i.e., onto the solar wing) and three curved surfaces with different curvatures (with curvature radii of 14.5, 11.5, and 5.8 cm, respectively), as shown in Fig. 7a.

Figure 7b–d show the changes in the output signals (including frequency, amplitude, and phase) of flexible SAW sensors under different UV intensities in four different states on the surface of the spacecraft model. The results reveal that different deformation states lead to entirely different UV-frequency, UV-amplitude, and UV-phase responses for the same flexible SAW device. The linear fitting functions based on these three features (i.e., using prediction models 1-3) and the XGBoost machine learning model were used to predict the UV intensity of flexible SAWs under different states. The obtained results are shown in Fig. 7e, f. These output results indicate that the conventional single feature-based prediction method produced significant deviations on curved and flat surfaces. In contrast, the XGBoost machine learning model produced results that showed much better consistency in the UV prediction of flexible SAW devices under four different bending states.

To quantify the relative errors using these four models in predicting the UV values of flexible SAWs on flat surfaces or under different bending states, the calibrated values at the maximum UV intensity were compared, and the output values of the four models correspond to different curvature states of the flexible SAW device. The results, summarized in Table 1, indicate that the traditional models based on single features have maximum relative errors exceeding 100%, whereas the model incorporating the XGBoost algorithm had a maximum relative error of 3.1% compared to the calibration value. These experimental results and model output results show good agreement, suggesting the substantial potential of integrated machine learning algorithms for accurately monitoring surface UV intensity using flexible SAW sensors in applications involving curved mechanical equipment or aerospace scenarios.

**Table 1:** Comparison of UV output values based on different models

Models UV (mW/cm²)	Model 1 (Frequency fitting)	Model 2 (Amplitude fitting)	Model 3 (Phase fitting)	Integrated XGBoost Model
Calibration Value	13.00	13.00	13.00	13.00
State 1 (flat)	12.68	13.23	10.44	13.05
State 2	10.05	22.77	2.36	13.07
State 3	7.5	23.14	-1.24	13.41
State 4	-0.5	27.60	-6.35	13.07
Maximum relative error	103.8%	112.3%	148%	3.1%

We compared the performance of our developed SAW UV sensors with those from the literature, as listed in Table S1. The UV sensing performance of our flexible SAW device is comparable to the sensing capabilities of most existing SAW UV sensors. However, our flexible SAW devices have a significant advantage over many of the previously developed rigid SAW UV sensors due to their adaptability to curved surfaces. Moreover, our flexible UV sensor can address critical issues such as unreliability and inconsistency of sensing outputs under random bending conditions.

## Conclusions

In conclusion, this study fabricated AlScN thin films with a c-axis preferred orientation on ultrathin flexible glass substrates and fabricated flexible SAW devices. The strain and ultraviolet sensing characteristics of flexible SAW devices were investigated, and the influences of bending or deformation on the frequency shift during UV sensing were theoretically analyzed. Additionally, we proposed a new strategy for predicting target parameters such as UV intensity by establishing a regression model between response signal features and parameters using machine learning algorithms. This strategy effectively minimizes the influence of dynamic bending strain on the sensing performance of flexible SAW devices, enabling accurate prediction of ultraviolet intensity under dynamic strain. Our results showed that the model based on the XGBoost algorithm presented the best UV (target) predictive performance under a strain interference of 0–1160 με, with a coefficient of determination of 0.996 and a normalized root mean square error of 0.019. To demonstrate the potential influence of our technology, flexible SAW sensors were adhered at four different and random positions on the curved surface of the spacecraft model, including a flat state and three curved surfaces with curvature radii of 14.5, 11.5, and 5.8 cm. The flexible SAW sensors showed highly reliable and consistent UV sensing performance under randomly curved conditions, with a maximum relative error of only 3.1% compared with those obtained on a flat surface. This study provides an effective solution for achieving reliable, consistent, and high-precision sensing by flexible SAW sensors, avoiding strain or deformation interference.

### Supplementary information

Supporting Information
