# Supplementary material for: Achieving consistency of flexible surface acoustic wave sensors with artificial intelligence
Source: Microsyst Nanoeng. 2024 Jul 5;10:94. doi: 10.1038/s41378-024-00727-z (PMC11226427; doi:10.1038/s41378-024-00727-z)
Supplement: Supplementary file 1 — Supporting Information [file 41378_2024_727_MOESM1_ESM.docx]

**Supporting Information**

**Achieving Consistency of Flexible Surface Acoustic Wave Sensors with Artificial Intelligence**

*Zhangbin Ji*^1^, *Jian Zhou*^1,*^, *Yihao Guo^1^*, *Yanhong Xia*^1^, *Ahmed Abkar*^1^, *Dongfang Liang^2^, Yongqing Fu*^3^

**AFFILIATIONS**

1. *College of Mechanical and Vehicle Engineering, Hunan University, Changsha 410082, China*
2. *Department of Engineering, University of Cambridge, Trumpington Street, Cambridge, CB2 1PZ, United Kingdom*
3. *Faculty of Engineering and Environment, Northumbria University, Newcastle upon Tyne, NE1 8ST, United Kingdom.*

**Corresponding E-mail:** jianzhou@hnu.edu.cn

1. **Figures**

**
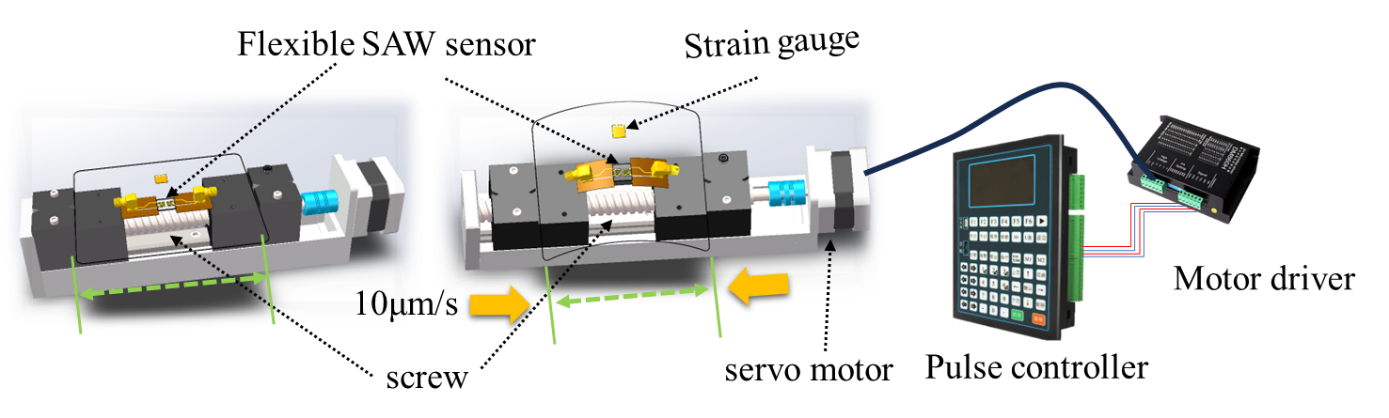
**

**Figure S1** Apparatus for applying dynamic strain to flexible surface acoustic wave devices.


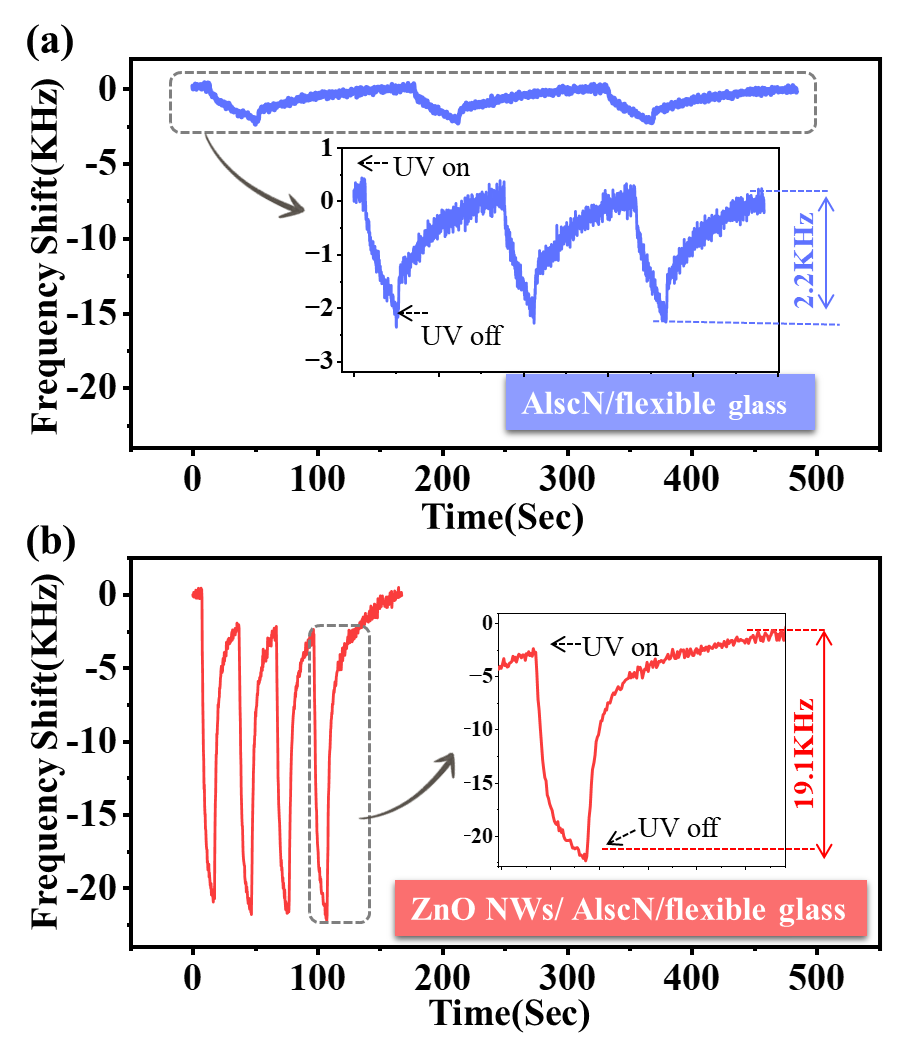


**Figure S2** (a) UV-frequency response characteristics of AlScN/flexible glass SAW device; (b) UV-frequency response characteristics of AlScN/flexible glass SAW device with a ZnO nanowire sensitive layer.


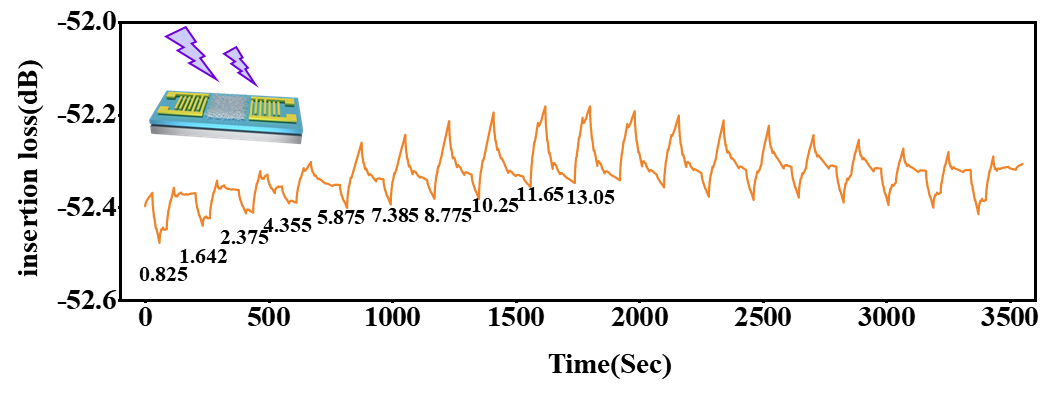


**Figure S3** Changes of insertion loss for flexible SAW sensor with continuous changes of UV power densities from 0.825 mw/cm^2^ to 13.05 mW/cm^2^.


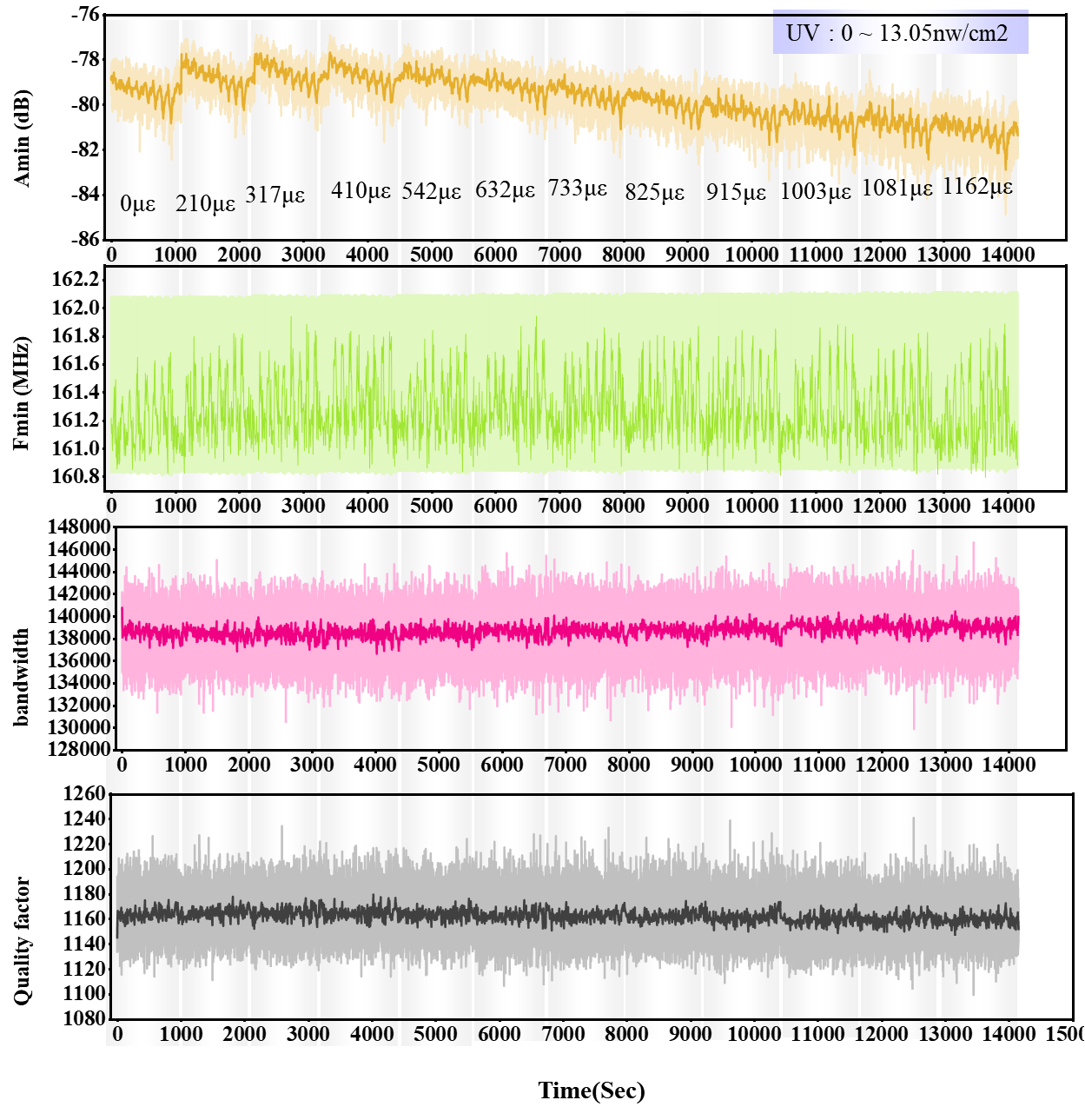


**Figure S4** Signal responses of SAW devices under different UV light intensities and strain states, including minimum amplitude (A_min_), minimum cutoff frequency (F_min_), bandwidth (BW), and quality factor (Q).


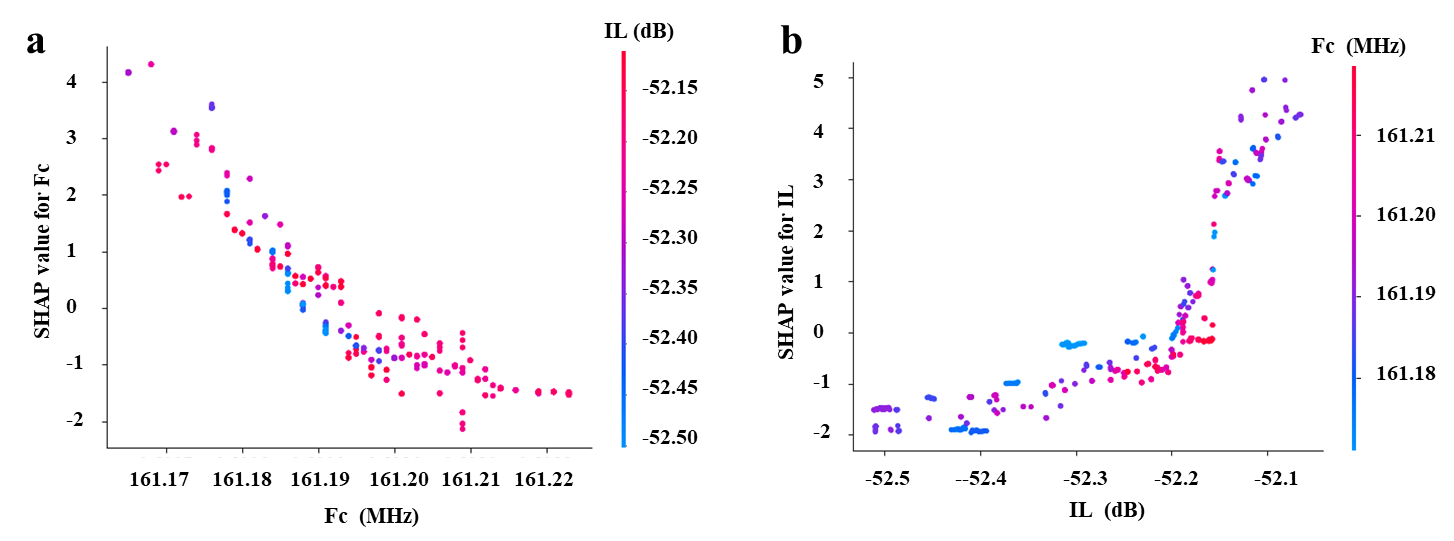


**Figure S5** Influences of interactions between two most important variables (i.e., center frequency Fc and insertion loss IL) on the prediction of ultraviolet intensity under a dynamic strain.


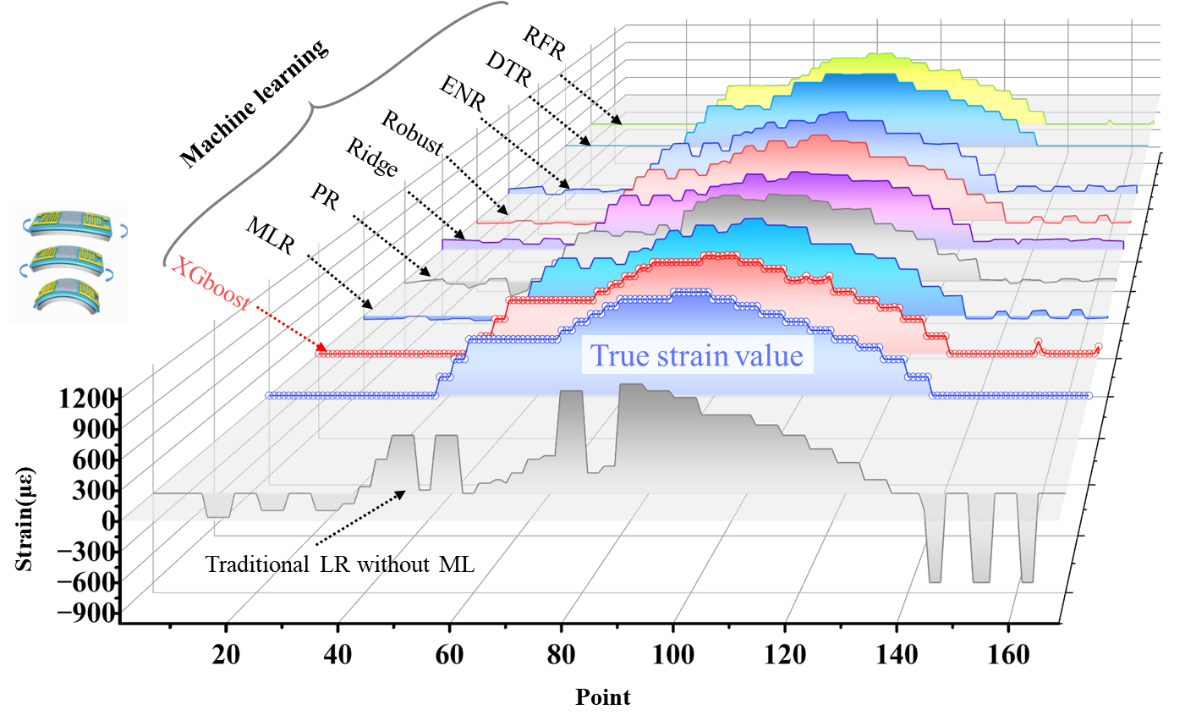


**Figure S6** Comparisons of predicted strain sensing results between conventional linear regression and machine learning models under the UV disturbance.

1. **Introduction to the Regression Algorithms Used**

**Multiple Linear Regression (MLR):** This is a foundational algorithm which models the relationship between a dependent variable and multiple independent variables by fitting a linear equation to the observed data. It assumes a linear relationship which the errors are normally distributed with constant variances.

**Polynomial Regression (PR):** Being an extension of MLR, polynomial regression fits a nonlinear relationship between the independent and dependent variables by introducing polynomial features. It can capture more complex patterns but is susceptible to overfitting if the degree of the polynomial is too high.

**Ridge Regression:** Also known as the L2 regularization, this ridge regression introduces a penalty term to the sum of squared residuals. This helps to prevent overfitting by shrinking the coefficients of less important variables towards zero, thus improving the model's generalizability.

**Robust Regression (Robust):** Designed to be less sensitive to outliers compared to the traditional regression methods, this robust regression uses techniques that reduce or ignore non-influential data points. This category includes methods such as Huber regression and M-estimation.

**Elastic Net Regression (ENR):** Combining both L1 (lasso) and L2 (ridge) regularization, elastic net regression balances the trade-off between feature selection and ridge's shrinkage. It can handle multicollinearity and is effective in high-dimensional data.

**Decision Tree (DTR):** A non-parametric method that models decisions as a series of binary decisions or "nodes." It is easily interpreted and visualized but can overfit if not pruned properly.

**Random Forest (RFR):** An ensemble method that builds multiple decision trees (a forest) and aggregates their predictions. It reduces the risk of overfitting and improves accuracy by introducing the diversity among the trees.

**Extreme Gradient Boosting (Xgboost):** To bring an advanced gradient boosting algorithm that scales tree methods, Xgboost is known for its speed and performance. It uses a combination of L1 and L2 regularization and can handle missing values, making it a versatile choice for various datasets.

These algorithms represent a range of approaches from the simple linear models to complex ensemble methods, each with their strengths and weaknesses. Selecting a specific certain model is dependent on the specific characteristics of the data and the major goals of the analysis.

1. **Table**

**Table S1.** Comparisons of UV detection performance of SAW devices from this study and those reported in literature.

| **Substrate** | **Substrate type** | **Anti-interference**  **of strain** | **Sensitivity (mw/cm^2^)^−1^** | **Response/**  **Recovery Time** | **Ref.** |
| --- | --- | --- | --- | --- | --- |
| ZnO/Al foil | Flexible | / | 3.89ppm | ~10s/~15s | 1 |
| LiNbO_3_ | Rigid | / | 9.53ppm | 15s/83s | 2 |
| ZnO/Quartz | Rigid | / | 19ppm | - | 3 |
| ZnO/Si | Rigid | / | 3ppm | - | 4 |
| ZnO/Si | Rigid | / | 0.6ppm | 5s/- | 5 |
| ZnO/flexible glass | Flexible | / | 1.66ppm | 8s/18s | 6 |
| AlN/flexible glass | Flexible | / | 2.8ppm | - | 7 |
| **AlscN/flexible glass** | **Flexible** | **Yes** | **10.6ppm** | **9s/16s** | **This work** |

**Reference**

1. Tao, X., et al., Three-Dimensional Tetrapodal ZnO Microstructured Network Based Flexible Surface Acoustic Wave Device for Ultraviolet and Respiration Monitoring Applications. ACS *Appl. Nano Mater*. 3, 1468-1478 (2020).

2.Guo, Y., et al., A new strategy to minimize humidity influences on acoustic wave ultraviolet sensors using ZnO nanowires wrapped with hydrophobic silica nanoparticles. *Microsyst Nanoen*g, 8,121 (2022)

3. Kumar, S., et al., ZnO based surface acoustic wave ultraviolet photo sensor. *J. Electroceram.* 22, 198-202 (2008).

4. Phan, D.T. and G.S. Chung, Characteristics of SAW UV sensors based on a ZnO/Si structure using third harmonic mode. Curr. Appl. Phys. 12, 210-213 (2012).

5. Guo, Y.J., et al., Ultraviolet sensing based on nanostructured ZnO/Si surface acoustic wave devices. Smart Mater. Struct. 24, 125015 (2015).

6. Yin, C.S., et al., Enhancing the sensitivity of flexible acoustic wave ultraviolet photodetector with graphene-quantum-dots decorated ZnO nanowires. Sens. Actuators, A. 321, 112590 (2021).

7. Ji, Z., et al., Flexible thin-film acoustic wave devices with off-axis bending characteristics for multisensing applications. Microsystems & Nanoengineering, 7,97, (2021).
